# Oncofetal Chondroitin Sulfate: A Putative Therapeutic Target in Adult and Pediatric Solid Tumors

**DOI:** 10.3390/cells9040818

**Published:** 2020-03-28

**Authors:** Nastaran Khazamipour, Nader Al-Nakouzi, Htoo Zarni Oo, Maj Ørum-Madsen, Anne Steino, Poul H Sorensen, Mads Daugaard

**Affiliations:** 1 Department of Urologic Sciences, University of British Columbia, Vancouver, BC V5Z 1M9, Canada; 2 Vancouver Prostate Centre, Vancouver, BC V6H 3Z6, Canada; 3 Department of Pathology and Laboratory Medicine, University of British Columbia, Vancouver, BC V5Z 1M9, Canada; 4 Department of Molecular Oncology, British Columbia Cancer Research Centre, Vancouver, BC V5Z1L3, Canada

**Keywords:** oncofetal chondroitin sulfate, chondroitin sulfate, cancer, solid tumors, target, pediatric cancer, VAR2

## Abstract

Solid tumors remain a major challenge for targeted therapeutic intervention strategies such as antibody-drug conjugates and immunotherapy. At a minimum, clear and actionable solid tumor targets have to comply with the key biological requirement of being differentially over-expressed in solid tumors and metastasis, in contrast to healthy organs. Oncofetal chondroitin sulfate is a cancer-specific secondary glycosaminoglycan modification to proteoglycans expressed in a variety of solid tumors and metastasis. Normally, this modification is found to be exclusively expressed in the placenta, where it is thought to facilitate normal placental implantation during pregnancy. Informed by this biology, oncofetal chondroitin sulfate is currently under investigation as a broad and specific target in solid tumors. Here, we discuss oncofetal chondroitin sulfate as a potential therapeutic target in childhood solid tumors in the context of current knowhow obtained over the past five years in adult cancers.

## 1. Oncofetal Similarities between the Fetal and Tumor Tissue Compartments

The placenta, an organ that develops during pregnancy, behaves in many ways like a tumor. In just 40 weeks, the placenta has to grow to a mass of ~500 grams, invade neighboring tissue, establish an elaborate vasculature, and escape the immune system, all key features of solid tumor development [1]. Moreover, similarities between placenta and cancer at the molecular level have been frequently observed. Several proto-oncogenes involved in malignant transformation and cancer progression, including c-*erbB1* family (*HER1*, *ERBB1* or *EGFR*), c-*myc*, *Fos* and c-*ras*, are preferentially expressed by trophoblast cells during the first week of pregnancy when the proliferative, migratory and invasive properties of these cells are at their peak [2,3]. For instance, c-*erbB1* is expressed exclusively by the cytotrophoblast in four- to five-week placentas and pre-dominantly in the syncytiotrophoblast compartment after six weeks of gestation [4,5,6]. It is also involved in the pathogenesis of numerous malignancies, including breast cancer [7] and some types of childhood cancer [8]. The c*-myc* (MYC) proto-oncogene displays strong expression in early placenta [9] and is also frequently increased in human cancers [10,11]. Hyperactivation of Ras signaling by mutations or overexpression of the *Ras* oncogenes is a powerful driver of solid tumor formation [12,13], and the *c-ras* proto-oncogene, a key player in signaling pathways that regulate cellular proliferation [14], is expressed in early villous trophoblasts [15,16]. Similarly, overexpression of the *Fos* proto-oncogene stimulates trophoblast invasion during placental implementation [17], while contributing to tumor metastasis in several types of cancer [18,19,20].

In addition to the expression of proto-oncogenes, a number of oncofetal proteins are also shared between placenta, tumors and fetal tissue, including pregnancy-associated plasma protein A (PAPP-A), PEG10, alpha-fetoprotein (AFP), carcinoembryonic antigen (CEA), trophoblast glycoprotein precursor (TPBG) and immature laminin receptor protein (iLRP). Based on their oncofetal properties, some of these proteins have since been pursued as potential therapeutic targets in solid tumors. For example, PAPP-A, which is produced by placental syncytiotrophoblasts and is essential for normal fetal development [21], has been shown to facilitate tumor growth and invasion in various malignancies [22]. Notably, PAPP-A has been investigated as a potent immunotherapeutic target in Ewing sarcoma [23]. Likewise, PEG10, an RNA splice factor that is crucial for placental and embryonic development [24], is reported to play a role in the progression of several types of human cancers, including leukemia, breast cancer, prostate cancer and hepatocellular carcinoma [25,26,27], and has been proposed as a therapeutic target for prostate cancer [26,27,28].

AFP is produced by the embryo during fetal development and is found in both fetal serum and amniotic fluid and is currently the most widely used prognostic marker in hepatocellular carcinoma [29,30]. Additionally, CEA produced during embryonal and fetal development is one of the most widely used tumor markers worldwide, especially in colorectal malignancies where it is used to detect and inform on the presence of liver metastasis [31]. In addition, TPBG is used as a prognostic tool in a broad spectrum of malignancies, including colorectal, ovarian and gastric cancers [32,33,34]. It is also the target of the cancer vaccine TroVax, currently in clinical trials for the treatment several solid tumor types [35,36,37,38]. iLRP, which is highly expressed in early fetal development, is re-expressed in many tumor types and has been associated with tumor progression and metastasis [39,40]. Moreover, iLRP has been investigated as a therapeutic target for patients with leukemic diseases and against metastatic spread of solid tumors [41]. There are thus numerous examples of oncofetal proteins that can be utilized as tumor targets.

To qualify as a tumor target, a protein must be differentially expressed between malignant and normal tissues. Inadequate differential expression of potential target proteins is a major concern for all targeted therapy approaches and there is therefore a high demand for discovery of new molecular targets, differentially expressed in malignant versus normal tissue. Post-translational modifications (PTMs) of proteins, including phosphorylation, glycosylation, ubiquitination, nitrosylation, methylation, acetylation, lipidation and proteolysis, increase the diversity of the proteome and influence almost all aspects of cell biology and pathogenesis [42]. Protein glycosylation has major effects on protein folding, conformation, distribution, stability and activity [43,44,45,46,47]. Given its critical role in expanding protein functionality and diversity, glycosylation is an attractive candidate source of molecular targets in cancer. Indeed, targeting the glycosylation component of a protein rather than the protein itself has clear advantages. Firstly, targeting of tumor-specific protein glycoforms could be a solution for increasing anti-tumor specificity while limiting off-target effects. Secondly, a specific glycosylation moiety or pattern can be present on several different proteins simultaneously across cell populations, including tumor stem cells, which may overcome challenges related to tumor heterogeneity and dormancy. Lastly, proteins that are not normally glycosylated may be subject to disease-specific glycosylations, thereby increasing the available tumor target reservoir [48,49,50].

## 2. Chondroitin Sulfate

Among the glycosylation components that play a critical role in protein functionality are glycosaminoglycans (GAGs). GAGs are large, linear, negatively-charged polysaccharides consisting of repeating disaccharide units that can be sulfated at different positions and to different extents [51,52]. Five GAG chains have been identified to date: Heparan sulfate (HS), chondroitin sulfate (CS), dermatan sulfate (DS), and keratan sulfate, as well as the non-sulfated hyaluronic acid [51,52]. GAGs are expressed on virtually all mammalian cells and are usually covalently attached to proteins, forming proteoglycans (PG).

CS is the second most heterogenous GAG group after HS and functionally presented as CS proteoglycans (CSPGs) in the pericellular matrix, as well as the intracellular milieu and the extracellular matrix (ECM) [53,54,55,56]. CS interacts with multiple ligands, both soluble and insoluble, and modulates important roles in many physiological and pathophysiological processes [57,58]. CS consists of repeating N-acetylgalactosamine (GalNAc)-glucuronic acid (GlcA) disaccharide units. A complex biosynthetic machinery in the Golgi apparatus is responsible for the production and structure of CS chains [59]. Five enzymes catalyze a tetrasaccharide-linker region attached to a serine residue of the core protein and six additional CS enzymes produce the polymeric backbone. During elongation of the CS chain, the sulfation of hydroxyl groups in different positions can occur. CS may contain sulfate groups in both the carbon 4 (C4) and C6 positions of the GalNAc unit (CSE), but may also be predominantly C4-sulfated (CSA) or C6-sulfated (CSC). Four CS carbohydrate sulfotransferases (CHSTs: CHST11, CHST12, CHST13 and CHST14) can catalyze the 4-O-sulfation of GalNAc in CS [60]. The CHSTs involved in 6-O-sulfation of GalNAc include (CHST3, CHST7, CHST15). The GlcA unit can also be sulfated at the C2 position, giving rise to DS also known as CSB (4-sulfated GalNAc and 2-sulfated GlcA) and CSD (6-sulfated GalNAc and 2-sulfated GlcA) [61]. The role of CS modifications in cancer progression has been under investigation for decades. In solid tumors, CS participate in cell–cell and cell–ECM interactions that promote tumor cell adhesion and migration, thereby facilitating aggressive and metastatic behavior of malignant cells [62,63,64,65]. Increased production of CS is found in transformed fibroblasts and mammary carcinoma cells, where these polysaccharides contribute to cell proliferation, adhesion and migration [64,66,67]. Similarly during embryonic development, CS in the context of CSPGs has important morphogenetic functions, especially in relation to epithelial morphogenesis, cell migration and cell division rates [68,69,70,71]. Moreover, CS is indispensable for pluripotency and differentiation of embryonic stem cells [72]. The ECM of human placentas contain high levels of CSPGs [73]. Placental CSPGs are mainly located on trophoblast cells in the ECM surrounding the expanding syncytium [63], where they are involved in a number of physiological processes. For example, they are part of a glycocalyx double-barrier that prevents the migration of immune cells through the placenta, from the mother to the offspring [72,74].

## 3. Oncofetal Chondroitin Sulfate in Placenta

In pregnancy-associated malaria pathogenesis, CSPGs in the placenta mediate the sequestration of infected red blood cells (IRBCs) to the intervillous spaces of the placenta [63]. Upon infection and during the replication phase inside IRBCs, the malaria parasite *Plasmodium falciparum* expresses a specific lectin, VAR2CSA, on the surface of the IRBCs. VAR2CSA subsequently binds to CS chains expressed in the placental syncytium, thereby enabling *P. falciparum* IRBCs to exit blood circulation and avoid filtration and destruction in the spleen of the infected host [75,76,77]. The specific form of CS recognized by VAR2CSA is a type of CSA [78,79] presented as a PTM on PGs such as syndecan-1 [63]. Evident by the fact that VAR2CSA-positive *P. falciparum* IRBCs sequester to the placenta as the only organ in the human host, placental CSA is thought to be distinct from CSA found in other tissues. Perhaps due to the phenotypical similarities between the placenta and tumors, placental-type CSA is also found in the vast majority of solid tumors as a secondary oncofetal CS (ofCS) PTM to PGs [80]. While the exact structure and composition of ofCS is as yet poorly understood, it is clear that the ofCS GAG chain is highly sulfated on C4 of the vast majority of GalNAc residues [80], and this specific sulfation pattern is unique to CSPGs in placenta and solid tumor tissue [80]. Since ofCS is not found in other normal tissues but the placenta, this PTM constitutes an attractive tumor target.

## 4. Expression of Oncofetal Chondroitin Sulfate Proteoglycans in Adult Solid Tumors 

Over the past five years, ofCS modifications of PGs have been described in multiple solid tumor indications [18,80,81,82]. Through binding and regulation of a large number of ligands, ofCS chains collaborate with other PG components to modulate cell behaviors such as proliferation, differentiation, migration and adhesion [63,80]. Although malignant tumors have individual CSPG profiles, they generally display strong ofCS expression [63]. Indeed, ∼90% of breast tumors, 80% of melanomas [80], and 92% of bladder cancers [82], express ofCS-modified CSPGs on the cell surface and/or in the tumor stroma. Moreover, ofCS alterations are often linked to disease progression and outcome in cancer patients. For example, expression of ofCS in melanoma tumors is significantly increased in advanced tumors, Clark level 2–5 compared to level 1, and in metastatic/recurrent disease compared to newly diagnosed disease [80]. In non-small cell lung cancer, high expression of ofCS correlates with poor relapse-free survival [80]. In addition, high ofCS expression is correlated with advanced tumor stage, cisplatin resistance and poor overall survival of muscle-invasive bladder cancer (MIBC) patients [82]. In breast cancer, CHST11 is over-expressed in tumors as compared to normal tissues [62]. Also, high expression of the CHST11 predicts poor disease-free survival of lung, breast and colorectal cancer patients [80]. Contrarily, other studies have reported that expression of C4-S sulfotransferases including CHST11 seems to be downregulated in colorectal cancers [83]. This discrepancy between different cancers highlights a lack of knowledge about the regulation and maturation of CS chains, which is further complicated by tissue-specific expression patterns and redundancy among CS enzymes. Nevertheless, ofCS expression is currently being evaluated as a potential therapeutic target for several adult tumor types, including bladder cancer [82], prostate cancer, breast cancer and non-Hodgkin’s lymphoma [80].

## 5. Expression of Oncofetal Chondroitin Sulfate Proteoglycans in Pediatric Solid Tumors 

While the expression of ofCS and its correlation with disease progression and outcome has been demonstrated in a variety of adult tumors, the potential for utilizing ofCS expression as a therapeutic target in childhood tumors has been less explored. Pediatric solid tumors are non-hematologic malignancies that occur during childhood. This heterogeneous group of tumors represents approximately 40%–50% of all pediatric cancers [84]. The tumor distribution of malignant pediatric solid tumors in adolescents is different compared with that of younger children, in whom embryonal or developmental cancers, such as retinoblastoma, neuroblastoma, or hepatoblastoma, are more prevalent. The most common malignant solid tumors in adolescents are extracranial germ cell tumors, bone and soft tissue sarcomas, melanoma, and thyroid cancer [85]. Generally, the outcome for pediatric solid tumors depends on location of the specific disease and risk group such as histological finding, tumor stage and metastatic status.

Similar to adult tumors, childhood solid tumors express various CSPGs with diverse functions related to disease progression (Table 1). In osteosarcoma, versican upregulation promotes cell motility and correlates with disease progression [39]. In neuroblastoma (NB), the CSPG NCAN is highly expressed in the tumor ECM where it facilitates growth of NB cells and promotes disease progression [82]. Exogenous NCAN expression transforms adherent NB cells into spheroids with high malignancy potential both in vitro (anchorage-independent growth and chemoresistance) and in vivo (xenograft tumor growth) [82]. CSPG4 is a cell surface PG commonly modified with ofCS that has been exploited as a tumor target in several tumor indications [86,87,88,89]. High levels of CSPG4 are found on a variety of adult and pediatric solid tumors including melanoma [90,91], osteosarcoma [87], rhabdomyosarcoma [88] and some brain tumors [86,92]. The CSPG4 expression levels differ depending on tumor type but is often present in both high-grade and lower-grade pediatric brain tumors [93]. PTPRZ1 plays a key role in cell migration, and is a potential tumor target in glioblastoma multiforme (GBM) [94]. In Ewing sarcoma, overexpression of APLP2 results in lower sensitivity to radiotherapy-induced apoptosis and immunologic cell death [95].

Proteoglycans can harbor different and multiple GAGs at the same time. For instance, syndecans and glypicans are PGs containing both CS and HS chains [96]. Altered expression of these PGs has been reported in multiple cancers including pediatric tumors [97]. Glypican 3, for example, plays an important role in cellular growth and differentiation. It is absent or only minimally expressed in most adult tissues but highly expressed in a variety of non-central nervous system (CNS) pediatric tumors, including hepatoblastoma, Wilms tumor, rhabdomyosarcoma, and in atypical teratoid rhabdoid tumors [98,99]. Glypican 5 is expressed in rhabdomyosarcoma where it facilitates growth factor signaling, in particular FGF signaling [100]. High syndecan-1 levels are found in glioma, where it correlates with advanced clinicopathological features and poor patient survival [101]. Sarcomas commonly express ofCS chains in 50%–100% of cases, depending on subtypes. Overall, ∼80% of bone sarcomas, and ∼85% of soft-tissue sarcomas are positive for ofCS [80]. Pediatric sarcoma cell lines generally express high levels of ofCS, and ofCS is required for migration and invasion capacity of osteosarcoma and rhabdomyosarcoma cells [80,102]. Indeed, ofCS has also been found on pediatric glioma cells and circulating tumor cells (CTCs) from GBM patients [89], hinting that ofCS might be exploited for liquid diagnostic applications in pediatric brain cancers. Also, ofCS allows for EpCAM-independent detection of CTCs [81], which might provide access to circulating sarcoma cells. Combined, the broad expression of CSPGs and ofCS across multiple pediatric tumor indications, promotes ofCS as putative and attractive therapeutic target in pediatric solid tumors.

**Table 1:** Chondroitin sulfate proteoglycan (CSPG) expression in childhood solid tumors.

CS-Modified PG	Cancer Type	Function
NCAN	Neuroblastoma	Promotes cell division, undifferentiated state and malignant phenotypes [82] Provides a growth advantage to cancer cells [82]
Versican	Osteosarcoma [103] Glioblastoma multiforme (GBM) [89]	Involves in TGFß - induced cell migration and invasion [103] Relevant marker of osteosarcoma progression [103] Potential target in cancer treatment [103] Function is unknown in GBM [89]
Decorin	Osteosarcoma [104]	Necessary for MG63 cell migration [104] Counteracts the growth-limiting effects of TGF-β2 [104]
CSPG4	Osteosarcoma [87] Rhabdomyosarcomas (RMS) [88] Medulloblastoma [105] Neuroblastoma [105] Childhood diffuse intrinsic pontine glioma [86] GBM [86,89] Dysembryoplastic neuroepithelial tumors (DNETs) [86,93]	Correlates with shorter survival in osteosarcoma [87] Therapeutic option for the combination treatment of RMS [88] Potential target for immunotherapy [87,89,105] Impairs terminal differentiation [86] Increases the invasive and migratory capabilities of glioma cells by facilitating interactions with extracellular matrix proteins [86] Facilitates angiogenesis by sequestering angiostatin [86] Increases tumor growth [86] Potential therapeutic target for treating childhood CNS cancers [86,89]
CD44	GBM [89,106]	High CD44 expression identifies GBM with particular poor survival chance Promotes aggressive GBM growth [106]
PTPRZ1	GBM [89,94]	Potential anti-cancer targets in GBM [89,94] Plays critical role in GBM cell migration [94]
APLP2	GBM [89] Ewing sarcoma [95]	Function is unknown in GBM [89] Anti-apoptotic function within Ewing sarcoma cells [95]
Syndecan-1	Glioma [89,101]	Correlates with the advanced clinicopathological features and lower survival rate [101]
Glypican 3	Hepatoblastoma Wilms tumor Rhabdomyosarcoma Atypical teratoid rhabdoid tumors	Potential candidate for targeted therapies [98]
Glypican 5	Rhabdomyosarcoma	Facilitates growth factor signaling Increases cell proliferation Potential target for therapeutic approaches [100]
Testican-1	GBM [89]	Unknown
Neuropilin-1	Osteosarcoma [107] Neuroblastoma [108] GBM [89]	Regulates metastasis potency [107] Correlates with poor response to chemotherapy [107] Correlates with poor prognosis for osteosarcoma patients [107] Regulates angiogenesis [107,108] Increases tumor growth [108] Function is unknown in GBM [89]

## 6. Oncofetal Chondroitin Sulfate as a Therapeutic Target in Solid Tumors

As outlined above, ofCS has emerged as an attractive tumor target for both therapeutic and diagnostic applications [18,80,81,82]. VAR2CSA specifically recognizes and binds ofCS, and recombinant VAR2CSA (rVAR2) proteins have been utilized to probe and access the ofCS chain expressed in solid tumors [80,82]. rVAR2 has also been exploited as a delivery system to shuttle cytotoxic drugs directly into ofCS-expressing tumor cells. For example, rVAR2-DT, a recombinant protein drug consisting of the cytotoxic domain of diphtheria toxin (DT388) fused to rVAR2, is able to eliminate both epithelial and mesenchymal tumor cells without any deleterious effect to normal primary human endothelial cells (HUVEC) in vitro [80]. Moreover, rVAR2-DT can inhibit prostate tumor growth in xenograft mouse models [80]. However, because DT-fusion drugs historically have shown adverse toxicity in human clinical trials [80], other strategies for delivery of drugs to ofCS-positive tumors have been pursued, including a rVAR2 drug-conjugate, VDC886. VDC886 is comprised of a 72 kDa rVAR2 polypeptide conjugated with the hemiasterlin toxin analog KT886, derived from the marine sponge *Hemiasterella minor*. VDC886 contains an average payload of three KT886 toxins per rVAR2 protein and displays strong toxicity towards diverse tumor cell lines of both adult and pediatric origin [80]. In vivo, VDC886 significantly inhibits tumor growth and metastasis in non-Hodgkin’s lymphoma, prostate cancer, and breast cancer xenograft models with no sign of adverse effects [80]. In a different study, VDC886 successfully targeted ofCS on cisplatin-resistant MIBC cells and suppressed tumor growth of MIBC in vivo [82]. Immunohistochemical analysis of two independent cohorts of matched pre- and post-neoadjuvant chemotherapy-treated MIBC patients, revealed that cisplatin-resistant residual tumors had elevated levels of ofCS expression, supporting ofCS as a marker for disease progression [82].

In summary, the broad expression of CSPGs across solid tumors, and of ofCS in particular, promotes ofCS as an attractive target for therapeutic intervention. Historically, targeted biologics-based therapies have been less successful in pediatric solid tumors as compared to adult cancers, largely due to low mutational burden and limited number of neoantigens [109]. Hence, targeting cancer-specific PTMs, such as ofCS, constitutes a novel opportunity to curb childhood solid tumors. Indeed, the ability of VDCs to target ofCS-positive solid tumors supports a rational for exploring additional ofCS-targeting strategies, such as chimeric antigen receptor (CAR) T cells and bi-specific immune-engagers (BiTEs).
